# Supplementary material for: Competitive exclusion during co-infection as a strategy to prevent the spread of a virus: A computational perspective
Source: PLoS One. 2021 Feb 24;16(2):e0247200. doi: 10.1371/journal.pone.0247200 (PMC7904198; doi:10.1371/journal.pone.0247200)
Supplement: S1 File — (PDF) [file pone.0247200.s001.pdf]

Supplementary material

Competitive exclusion during co-infection as a strategy to prevent the spread of a virus: a computational perspective

Safar Vafadar[1☯], Maryam Shahdoust[2☯], Ata Kalirad[2☯], Pooya Zakeri[3, 4☯] and Mehdi Sadeghi[2, 5*]

1 Laboratory of Biological Complex Systems and Bioinformatics (CBB), Institute of Biochemistry and Biophysics, University of Tehran, Tehran, Iran
2 School of Biological Science, Institute for Research in Fundamental Sciences (IPM), Tehran, Iran
3 Centre for Brain and Disease Research, Flanders Institute for Biotechnology (VIB), Leuven, Belgium.
4 Department of Neurosciences and Leuven Brain Institute, KU Leuven, Leuven, Belgium.
5 National Institute of Genetic Engineering and Biotechnology (NIGEB), Tehran, Iran

☯These authors contributed equally to this work.
* sadeghi@nigeb.ac.ir

A) Choosing the parameters of the model

To determine the range of the parameters value we ran different simulations, for a single virus model, by changing the parameter values and made different combinations of the different parameter values. Waiting times, the times that each virus spends in each stage of viral infection, were chosen from Exponential or Weibull distribution. To shorten the length time of one stage, we should increase the parameter λ of the Exponential distribution ($\lambda>1$) and decrease the shape parameter of the Weibull distribution ($\omega<1$). After running different simulations, we figured out that shortening the length of the viral infection process affects the number of infected cells (and produced virions) but the total trend is similar in all the simulations. Therefore, we decided to report just the simulation in which the parameter λ is 3 and the parameter ω is 0.5, in the main text of the manuscript.

The plot at the below displays the result of the different simulations due to changing the parameter of the Exponential distribution to generate the waiting times at the replication stage. In addition, the table next to the plot demonstrates the value of the probability distributions to generate waiting times at different stages of viral growth process.

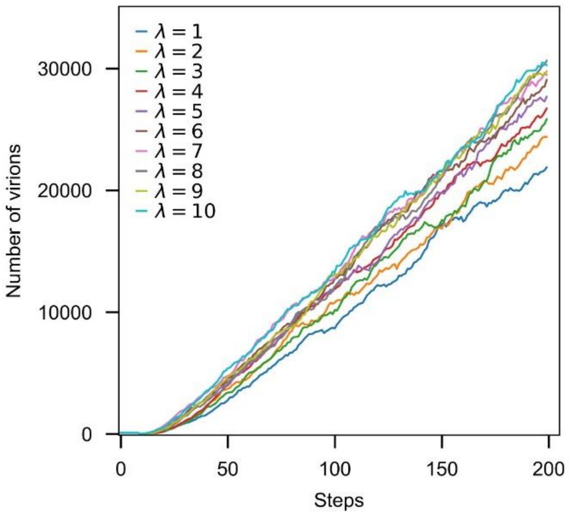

**Stage**	**Probability Distribution**
Attachment-Penetration	Exp (λ=1)
Penetration-Eclipse	Exp (λ=1)
Eclipse-Replication	Weibull(λ=1,ω=0.5)
Replication-Release	Exp(λ=1,… ,10)*

(*) Simulations repeated due to ten different values of the parameter λ.

B) Competition of two viruses during Co-infection in the model with active immune system

The plots at the below demonstrate the co-infection of virus B—the benign virus— and virus M—the malignant virus— at three conditions of co-infection delay (D= -30, D=0, D= +30) when the immune system was active.

We assumed four different modes for the immune system: weak and limited capacity (WL), weak and unlimited capacity (WU), strong and limited capacity (SL), strong and unlimited capacity (SU).

At the plots, the each trajectory is the average of 100 runs. The shaded areas contain the trajectories of all the 100 runs for the given parameters. For virus M, all the parameters of probability distributions to generate the waiting times were set as one. For virus B, the waiting times had been generated from Exp ($\lambda = 3$) and Weibull ($\lambda = 1$; $\omega = 0.5$). The plots show that the benign strain is dominant in all three conditions of co-infection delay (D).

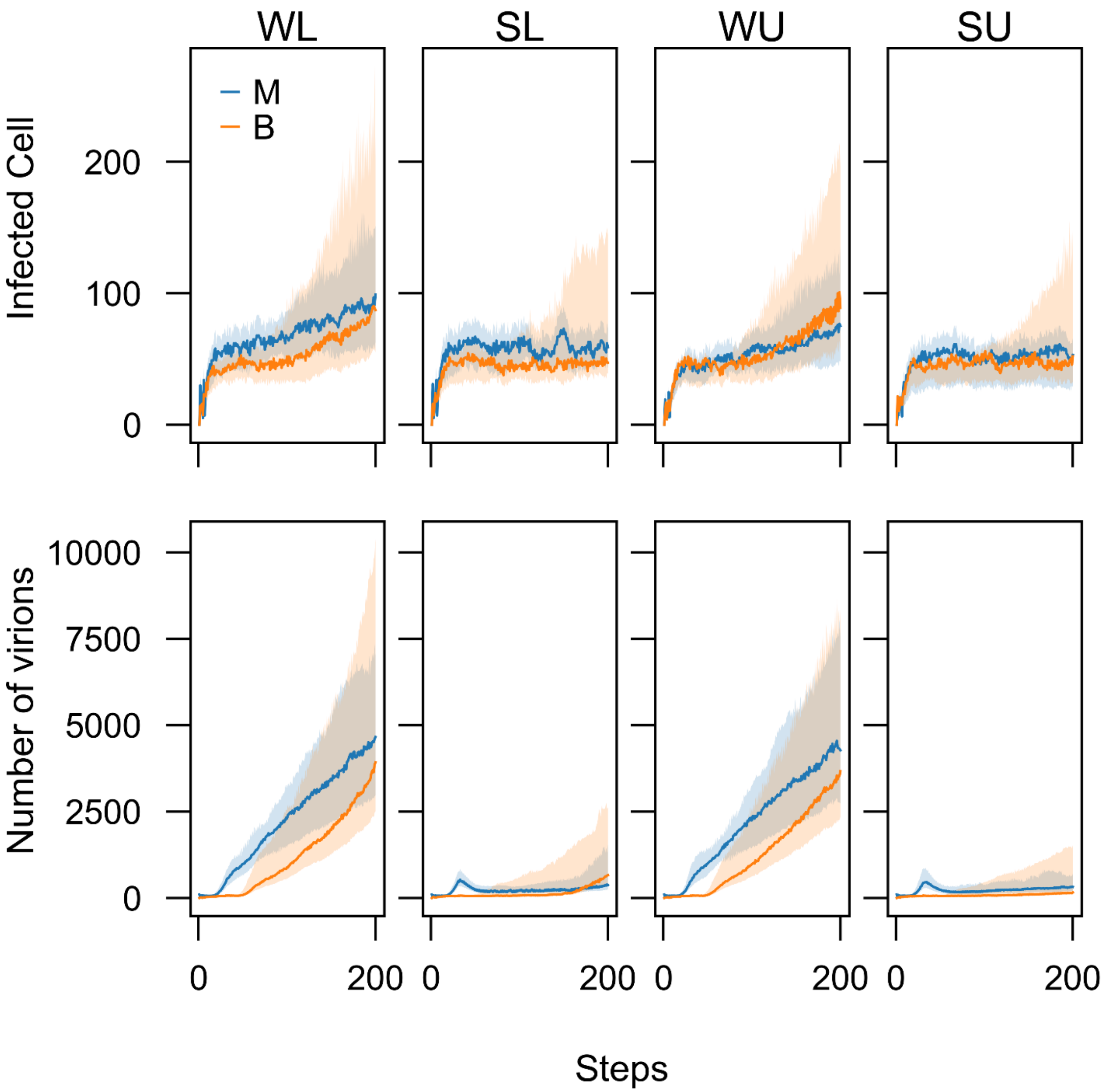

Supplementary Figure 4. Introduction of the benign strain (B) 30 steps after the introduction of the malignant strain (D = -30)

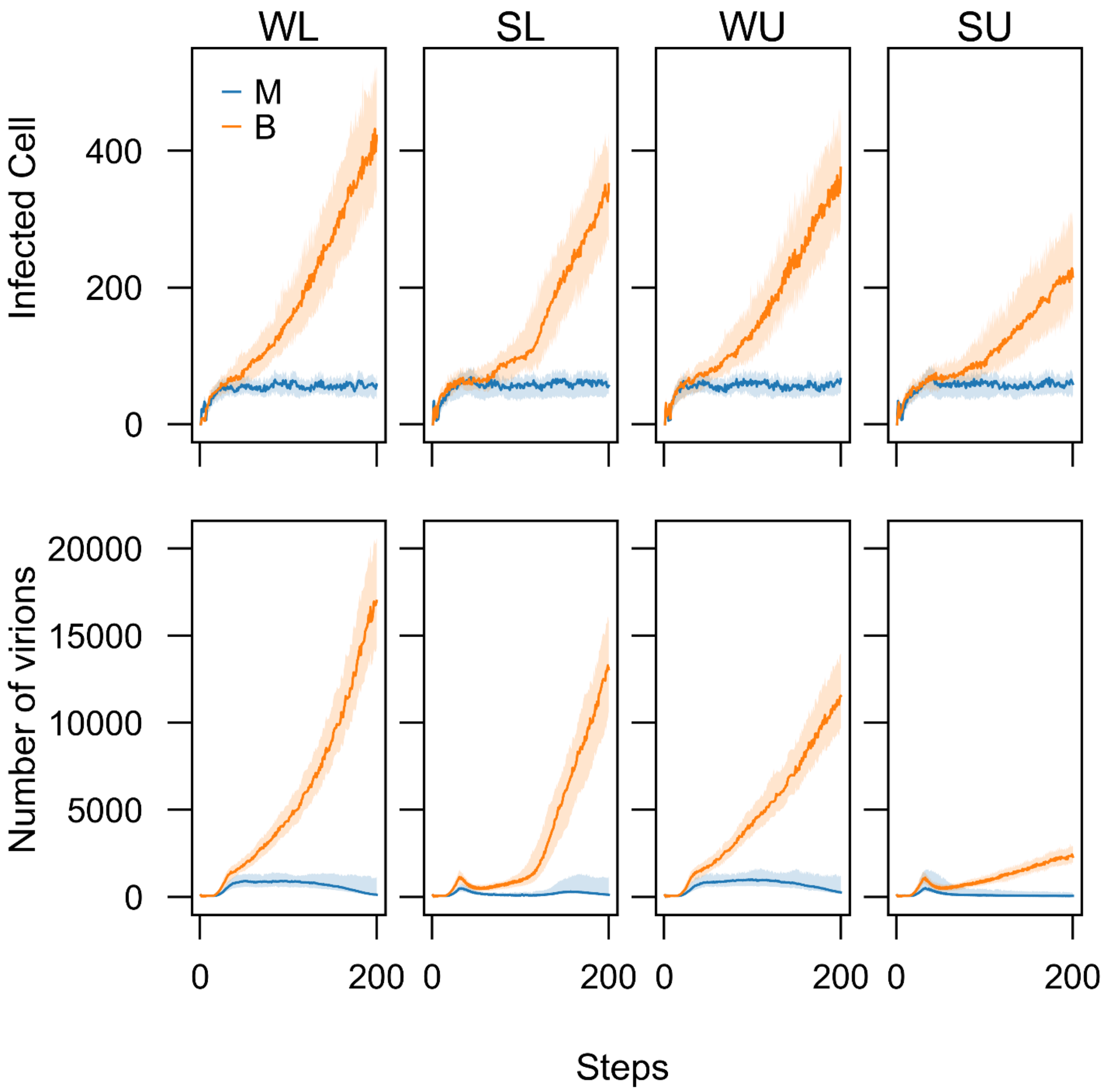

Supplementary Figure 5. Introducing the benign (B) strain and malignant strain (M) at the same time (D = 0)

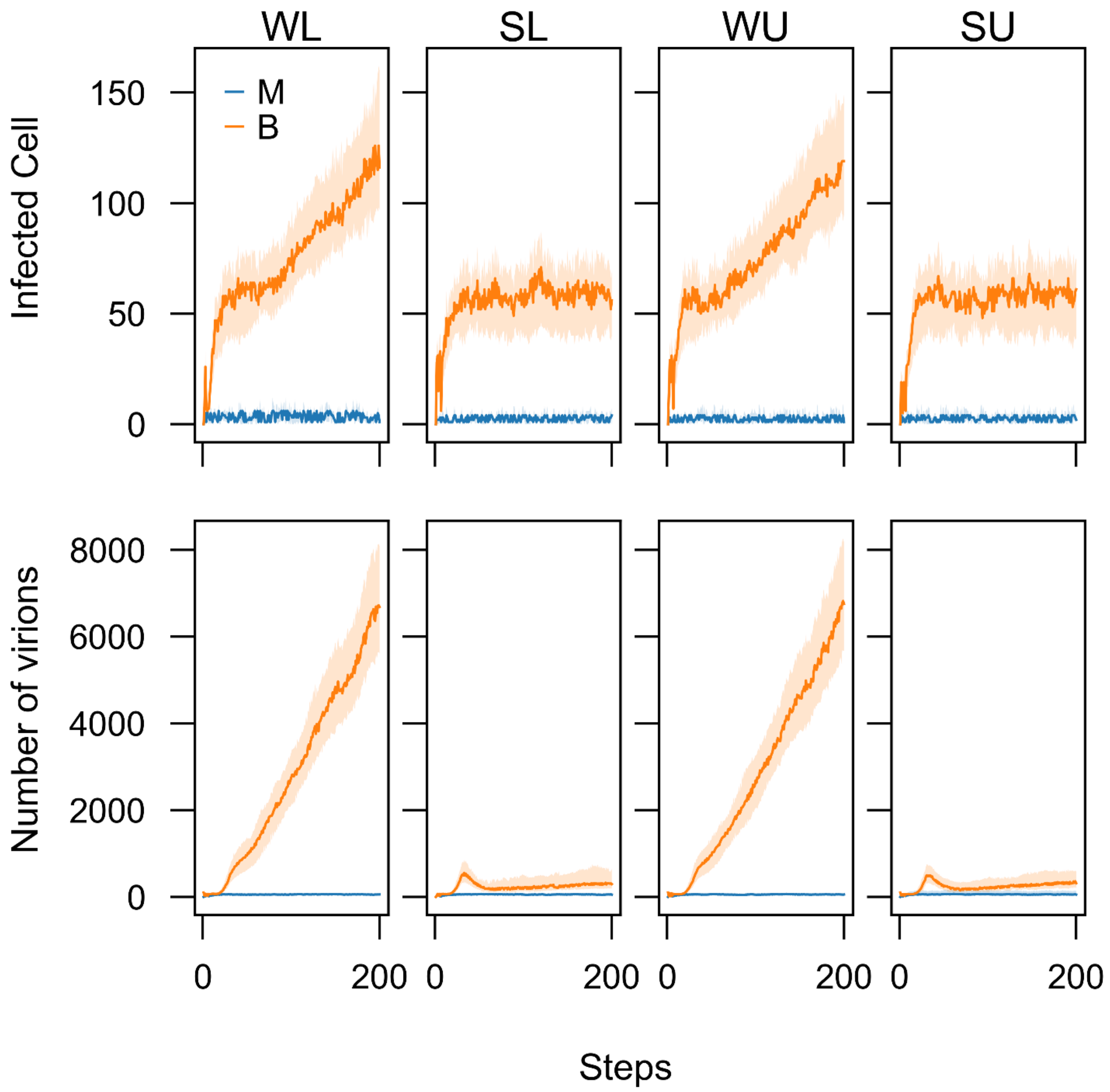

Supplementary Figure 6. Introduction of the benign strain (B) 30 steps before the introduction of the malignant strain (D = +30)
