# Supplementary material for: Competitive exclusion during co-infection as a strategy to prevent the spread of a virus: A computational perspective
Source: PLoS One. 2021 Feb 24;16(2):e0247200. doi: 10.1371/journal.pone.0247200 (PMC7904198; doi:10.1371/journal.pone.0247200)
Supplement: Attachment — Submitted filename: Response to Reviewer #3 comments.docx [file pone.0247200.s003.docx]

**Responses to Reviewer #3 comments**

Dear Prof. Bashar Ibrahim

We would like to thank you and the reviewers for consideration of the revised manuscripts and point-by-point responses. We are glad that most of the reviewers’ points in the previous revision were mostly met by the revised manuscript and our responses.

Please find enclosed our point-by-point responses to the concerns of Reviewer#3, Andrew Glover, as well as a revised version of our manuscript (with modifications tracked in green).

We hope that these changes will make the manuscript suitable for publication in your journal and remain available to receive any further comments on our answers.

Sincerely,

Mehdi Sadeghi,

On behalf of the authors

Reviewer #3: I would like to thank the reviewers for addressing the comments myself and the other reviewers had made in response to the manuscript's initial submission, and believe the presentation of the work has improved as a result. I do nevertheless have some ongoing concerns:

We would like to thank the reviewer’s suggestions and encouraging words. The responses to his comments are as follows:

C1) While we are thankful that the authors have given an improved explanation behind their choice of parameters in the supplementary information, we still believe that the paper would benefit significantly if these were placed in the context of some real world viruses. Currently the choice of parameters, both in the main body of the text and the supplementary information, are entirely theoretical and have not been justified. I feel this would be acceptable if the paper was presented as a wholly theoretical piece of work, however, the authors ultimately conclude that "Co-Wish suggests that common respiratory viral infections - such as common human coronaviruses - may limit the replication of SARS-CoV-2" (lines 317-318). Yet, when the choice of parameters is entirely theoretical, I do not believe such conclusions should be drawn, or even alluded to.

A1)

We followed your suggestion; accordingly, we have edited the manuscript to emphasize the theoretical aspects of the study.

In the Abstract of the revision, the text reads:

“While it could prove challenging to fully understand the therapeutic potentials of viral co-infection, we discuss that our theoretical framework hints at an intriguing research direction in applying co-infection dynamics in controlling any viral outbreak’s speed.”

In the Introduction of the revision, the paragraph below is now omitted:

“Based on the above assumptions, we propose that the competitive exclusion principle can, at least theoretically, be useful in tackling a viral pandemic, such as the current COVID-19 crisis, since one viral strain might be able to out-compete and eliminate a rival strain in the same ecological niche. Considering the current pandemic, this question offers hope as a means to control the novel coronavirus outbreak by supplanting the SARS-CoV-2 virus by a much less fatal one.”

The Discussion section has been edited as well; for example, some explanations about the SARS-CoV-2 virus is now deleted.

- At the beginning of the discussion, the following text is now dropped:

“We looked into the dynamics of viral co-infection to investigate the possibility of eliminating malignant virus strains in the population by pitting it against a more benign strain.”

The text lines [230]-[233] now reads:

“In this study, we look into the dynamics of viral co-infection using a CEP-inspired computational model. To explore the possible effects of co-infection by two virus species, we develop a stochastic-based viral infection simulator—called Co-Wish—to model and display the competition between two strains.”

- In the discussion, the paragraph below is now omitted:

“Following the same line of thought, we examined the possibility of slowing down the spread of COVID-19 through the introduction of a more harmless virus to a population. Our results offer the possible mitigating role of benign respiratory virus infection as a way of antiviral treatment in COVID-19 individuals. Accordingly, there is a clear and pressing need to conduct clinical trials to assess the role of co-infection in the clinical outcomes of SARS-CoV-2 infected patients.”

- Revised discussion focuses more on the theoretical aspects of the proposed models. We only discuss that our theoretical framework hints at an intriguing research direction in the dynamics of co-infection. Accordingly, the fifth and sixth paragraphs in the previous version are summarized into one paragraph—highlighted in green in the revised manuscript—which only concisely and suggestively discusses the possibility of controlling the spread of any viral infection, such as the SARS-CoV-2 virus, in the population by supplanting the lethal virus with a much less fatal one.

C2) The results of the paper are still presented over steps rather than units of time. While I appreciate the authors responded to this point when I raised it previously, I still feel that it would be important to translate the results to be presented over time units if conclusions such as the one highlighted above are to be drawn. Moreover, it should not be too difficult for the authors to record the sojourn times between events from a Gillespie algorithm.

A2)

Regarding the definition of one step in our simulation, **we present the results over almost equal-length segments of assumed time**. One step is terminated when the sum of the time length of different runs of the Gillespie algorithm equal to, at least, the determined time length of steps. In our simulation, the length of steps is about 1000msec; **we represent the results once every 1000msec.**

The summary of our reasons for displaying the results by using step number are as follows:

- Avoiding Computational Costs:

Using Discrete-Time Simulation (DTS), for example, displaying the results at each millisecond, imposes a lot of computational overhead such as CPU,IO, and RAM overhead. The computational costs make the simulation slow.

Instead, we apply the TIME ADVANCE MECHANISM (TAM) approach and a parallel timeline for each infected cell viral growth process.

- Preparing Concise view about the parallel simulations:

We believe that presenting the results over a number of steps can illustrate a more concise view about the parallel simulations held in different virtual infected cells than showing them over time. The main reason for this decision is simulations at infected cells are in parallel with different lengths of time. Showing the results over the number of steps allows the viral growth process to get finished in more infected cells. Figure 1 displays the main idea of the simulation.

Figure 2 shows the simulation results over time unit (millisecond) just for one run. In our study, each simulation includes one hundred runs. The trajectory is very dense because it represents a large number of points. It seems it does not convey more information than displaying the results over the number of steps.

Figure 1- schematic picture of viral infection simulation. Simulations in “n” cells are simultaneous. Different clocks illustrate different time lengths of the simulations. “Evaluate Time to Infect Neighbors” checks out the time length of the simulation.

Figure 2- simulation results over time unit (millisecond) just for one run
